# Abp1 promotes Arp2/3 complex-dependent actin nucleation and stabilizes branch junctions by antagonizing GMF

**DOI:** 10.1038/s41467-018-05260-y

**Published:** 2018-07-24

**Authors:** Siyang Guo, Olga S. Sokolova, Johnson Chung, Shae Padrick, Jeff Gelles, Bruce L. Goode

**Affiliations:** 1 Department of Biology, Brandeis University, Waltham, MA 02454 USA; 2 Department of Biology, Moscow M.V. Lomonosov University, Moscow, 119234 Russia; 3 Department of Biochemistry, Brandeis University, Waltham, MA 02454 USA; 4 Department of Biochemistry and Molecular Biology, Drexel University College of Medicine, Philadelphia, PA 19102 USA

## Abstract

Formation and turnover of branched actin networks underlies cell migration and other essential force-driven processes. Type I nucleation-promoting factors (NPFs) such as WASP recruit actin monomers to Arp2/3 complex to stimulate nucleation. In contrast, mechanisms of type II NPFs such as Abp1 (also known as HIP55 and Drebrin-like protein) are less well understood. Here, we use single-molecule analysis to investigate yeast Abp1 effects on Arp2/3 complex, and find that Abp1 strongly enhances Arp2/3-dependent branch nucleation by stabilizing Arp2/3 on sides of mother filaments. Abp1 binds dynamically to filament sides, with sub-second lifetimes, yet associates stably with branch junctions. Further, we uncover a role for Abp1 in protecting filament junctions from GMF-induced debranching by competing with GMF for Arp2/3 binding. These data, combined with EM structures of Abp1 dimers bound to Arp2/3 complex in two different conformations, expand our mechanistic understanding of type II NPFs.

## Abstract (web summary)

Abp1, a type II actin nucleation promoting factor, is a known component of branched actin networks but its mechanism remains poorly understood. Here, the authors find that Abp1 enhances Arp2/3-mediated actin branch formation, and blocks ‘debranching’ by GMF, making it a pro-branching factor.

## Introduction

The first actin nucleating factor discovered was the actin-related protein (Arp) 2/3 complex, which consists of seven evolutionarily conserved subunits and generates branched actin arrays that drive cell protrusion, motility, endocytosis, intracellular transport, and pathogen invasion and motility^1–3^. Despite the central biological importance of this actin nucleator, our understanding of how the Arp2/3 complex is spatially and temporally regulated in cells remains limited. The Arp2/3 complex alone exists in an inactive state, and requires binding of a nucleation-promoting factor (NPF) to trigger actin nucleation. NPFs fall broadly into two categories, type I and type II, which bind actin monomers vs. actin filaments, respectively^4^. Type I NPFs and type II NPFs are often found localizing to the same actin structures in vivo, and genetic evidence suggests that they both have important roles in controlling actin network assembly and turnover^5–7^. However, their functional and mechanistic differences are still being determined.

Type I NPFs, which include WASP, WAVE/SCAR, WASH, WHAMM, and JMY, have a VCA region consisting of a WASP-homology 2/Verprolin homology domain (V), a central or connecting sequence (C), and an acidic motif (A)^8,9^. In the VCA module, the A motif binds Arp2/3 complex and the V motif binds actin monomers. Efficient Arp2/3 activation requires interaction of two WASP molecules with separate binding sites on Arp2/3 complex, and in vivo WASP can be dimerized by various Src homology 3 (SH3) domain-containing binding partners^10–12^. VCA binding alters Arp2/3 complex conformation^13–16^, priming it for nucleation, and recruits actin monomers to the Arp2 and Arp3 subunits, generating a stable nucleation seed. Single-molecule analysis has further revealed that VCA priming of Arp2/3 complex causes a small increase in the effectiveness of filament side binding, and increases the likelihood of a bound complex nucleating a daughter branch^17^.

Type II NPFs, which include Cortactin and Abp1, bind to filamentous actin (F-actin) instead of actin monomers and show weaker nucleation-stimulating effects in bulk assays compared to type I NPFs^18–20^. Cortactin is conserved in animals but not yeast, and is required along with WASP for proper formation of podosomes, invadipodia, and other actin-based cellular structures^21^. Cortactin also stabilizes actin networks in vivo, in part by antagonizing Coronin-mediated turnover of branches^22^. Cortactin synergizes with WASP in stimulating Arp2/3-mediated actin nucleation in vitro, and has been suggested to recruit Arp2/3 complex to the sides of mother filaments, although this has not been directly visualized^19,20,23–26^. Cortactin has also been suggested to stimulate VCA release from nascent Arp2/3-actin seeds, which is a necessary step in branched nucleation^27,28^. Single-molecule analysis on Cortactin shows that it binds preferentially to branch sites, where it remains bound during daughter filament elongation^27^. Single-particle electron microscopy has been used to solve the structure of Cortactin–Arp2/3^29^, confirming earlier reports that Cortactin contacts multiple subunits in Arp2/3 complex and competes with VCA for binding to Arp3^23^. Three-dimensional (3D) reconstructions of actin filaments decorated by Cortactin further reveals that Cortactin alters F-actin conformation, possibly as a mechanism to recruit Arp2/3 to filament sides and/or stabilize branches^30^.

Abp1 is widely conserved across fungal and animal species, where it is a component of branched actin networks^31^. Abp1 was first identified in *Saccharomyces cerevisiae* on F-actin affinity columns^32,33^, and later characterized as an Arp2/3 regulator^18^. In yeast and mammalian cells, Abp1 localizes to sites of endocytosis, along with WASP, and makes important contributions to actin-dependent membrane invagination^34–39^. Abp1 contains an N-terminal actin depolymerization factor (ADF)-homology domain that binds F-actin and a C-terminal SH3 domain that binds several other proteins. In addition, yeast Abp1 contains two acidic motifs (A), located between the ADF-homology (ADFH) and SH3 domains, which are not present in vertebrate homologs of Abp1. Yeast Abp1 NPF effects depend on both its ADF-homology domain and its A motifs^18,40^, but to date its NPF activities have only been studied in bulk assays^18,40^.

In the absence of direct observations of Abp1's effects on actin filaments by total internal reflection fluorescence (TIRF) microscopy, its mechanism as an NPF has remained unresolved. Further, the potential effects of Abp1 on branch stability have never been addressed, which is relevant given that another ADF-homology protein, glia maturation factor (GMF), has been shown to bind Arp2/3 complex and catalyze debranching in vitro and in vivo^41–44^. Here we show that Abp1 enhances Arp2/3-mediated actin nucleation by stabilizing Arp2/3 complex binding to the sides of actin filaments. Abp1 dynamically interacts with actin filament sides, but stably associates with Arp2/3-actin branch junctions. At filament branch junctions, Abp1 can also use its ADFH domain to block GMF debranching effects on Arp2/3 complex. Further, Abp1 dimerizes upon binding Arp2/3 complex, and can adopt at least two distinct conformations, which lend insights into its type II NPF mechanism of regulating Arp2/3 complex.

## Results

### Abp1 stimulates Arp2/3-mediated branched actin nucleation

**Fig. 1:**
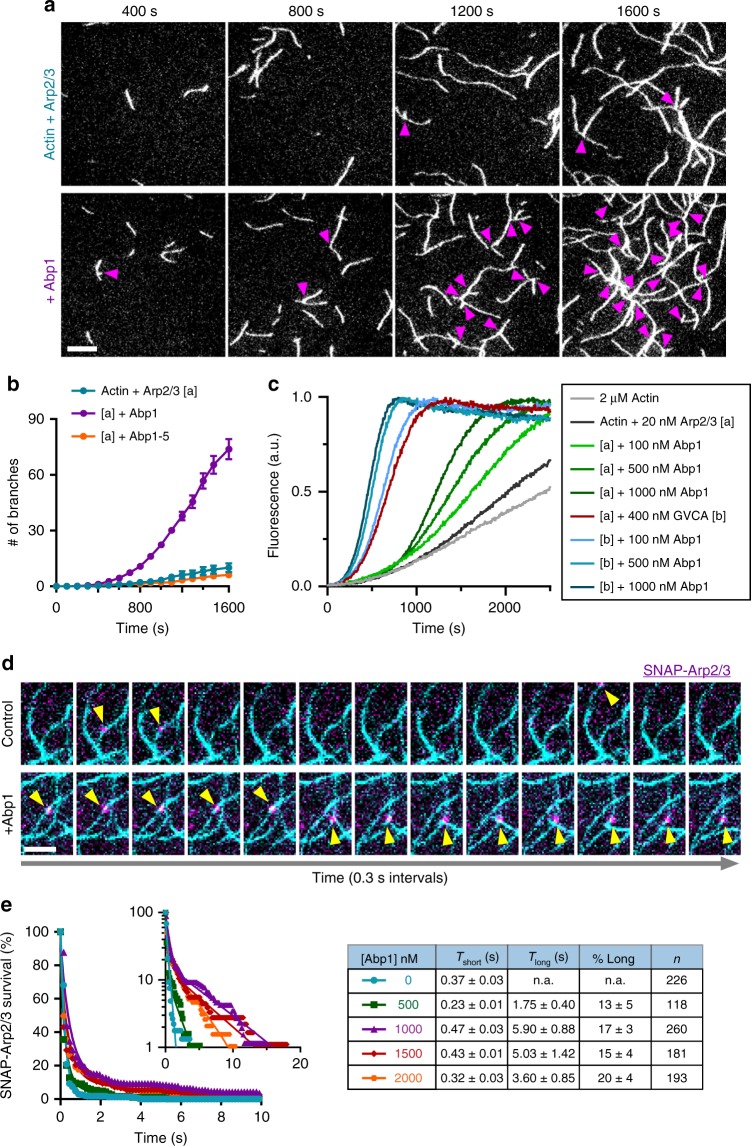
Abp1 stimulates Arp2/3-mediated nucleation of daughter branches. **a** Time points from representative TIRF microscopy actin assembly assays, with magenta arrows marking branch junctions. Reactions contain 1 µM actin (10% OG-labeled), 8 nM Arp2/3 complex ± 300 nM Abp1. Scale bar, 10 µm. **b** Kinetics of branch formation, quantified as the number of branches nucleated per FOV (18,000 µm^2^), averaged from 3 FOVs per reaction in each of two trials. Error bars, s.e.m. **c** Combined effects of Abp1 and GST-VCA on Arp2/3-mediated actin nucleation in pyrene actin assembly assays. Reactions contained 2 µM actin (10% pyrene labeled), 20 nM Arp2/3 complex, 400 nM GST-VCA (GVCA) and/or varying concentrations of Abp1, as indicated. **d** Montage showing association of JF646-SNAP-Arp2/3 complex molecules (10 nM, magenta) with anchored actin filaments (cyan), in the presence and absence of 1 µM Abp1, at 0.3 s intervals. Scale bar, 5 µm. **e** Cumulative survival curves (symbols) of JF646-SNAP-Arp2/3 molecules bound to actin filaments in the absence or presence of different concentrations of Abp1. Lines are fits to single exponential (absence of Abp1) or double exponential (presence of Abp1) distributions. Inset shows the same data, plotted on a log scale. Table includes number of binding events (*n*), fitted parameter values (*T*_short_, *T*_long_, and % long) with s.e. from *n* = 10,000 bootstraps samples

Until now, Abp1's effects on Arp2/3 complex have only been examined in bulk assays^18,40^. Therefore, we decided to reinvestigate Abp1's NPF activity using TIRF microscopy, where we could directly visualize and more accurately quantify daughter branch nucleation events, as well as filament elongation rates (Fig. 1a and Supplementary Figure 1a). In TIRF assays, we observed a near eightfold increase in the total number of branches nucleated by Arp2/3 complex per field of view (18,000 µm^2^) (Fig. 1b). Further, Abp1-5, which carries a point mutation in the ADF-homology domain of Abp1 that abolishes NPF effects in bulk assays^40^, also failed to stimulate nucleation in our TIRF assays (Fig. 1b). We considered whether the discrepancy between our TIRF results and earlier bulk assays might stem from additional (negative) effects of Abp1 on filament elongation, which would be masked in bulk assays. However, when we measured rates of elongation for mother and daughter filaments in our TIRF reactions, the presence of Abp1 had minimal effect (Supplementary Figure 1b). We also considered whether Abp1 might quench pyrene-actin in bulk assays, as ADF/Cofilin has been shown to have this effect^45^. Our direct tests confirmed that ADF/Cofilin strongly quenches pyrene-F-actin fluorescence (Supplementary Methods); in contrast, Abp1 did not (Supplementary Figure 1c). Therefore, it is more likely that the discrepancy arises from a higher background of spontaneous nucleation in bulk assays, where higher concentrations of actin are used. Regardless, in TIRF assays, where branch nucleation events are directly observed, Abp1 strongly enhanced Arp2/3-mediated nucleation.

In addition, we asked whether Abp1, a type II NPF, can stimulate Arp2/3-mediated actin nucleation when present with a canonical type I NPF, yeast WASP/Las17. We used bulk assays, since our dimeric VCA construct, GST-VCA, induced formation of large, dense clusters of actin filaments in TIRF assays, precluding accurate quantification of branched nucleation events. In pyrene actin assembly assays, GST-VCA alone strongly stimulated Arp2/3-dependent actin nucleation (Fig. 1c). Further addition of different concentrations of Abp1 enhanced nucleation by GST-VCA. Previous studies showed that a different type II NPF, Cortactin, can synergistically stimulate Arp2/3-mediated actin assembly with WASP^27^.

### Abp1 stabilizes Arp2/3 complex on actin filament sides

To better understand how Abp1 enhances actin nucleation, we used multi-wavelength single-molecule TIRF to visualize labeled Arp2/3 complex (JF646-SNAP-Arp2/3) interacting with preformed, unbranched actin filaments (in the absence of actin monomers) with and without Abp1 present (Fig. 1d). Consistent with earlier studies^17^, we found that Arp2/3 complex bound to filament sides very dynamically, with an average lifetime of 0.37 s (Fig. 1e). However, the presence of 0.5 μM Abp1 led to 13% of Arp2/3 binding events on filament sides lasting longer, ~2 s (Fig. 1e). At higher concentrations of Abp1 (1, 1.5, and 2.0 μM), the longer-lived Arp2/3 binding events increased to ~4-6 s, but the percentage of long-lived binding events did not increase beyond ~20% (Fig. 1e). This ability of Abp1 to stabilize only a fraction (~20%) of Arp2/3 complex interactions with filament sides raises the possibility that Abp1 stabilizes only specific conformations of Arp2/3 bound to filaments. Further, the ability of Abp1 to increase Arp2/3 lifetime on filament sides likely makes an important contribution to stimulating branch formation, given that Arp2/3 binding to mother filaments is otherwise very transient, and severely limits the fraction of binding events that result in branched nucleation^17^. Although such stabilizing effects have been hypothesized for NPFs, until now they have never been directly observed for any NPF, including WASP-VCA and Cortactin. Thus, our observations on Abp1 expand our understanding of the range of mechanisms used by NPFs to stimulate Arp2/3-mediated actin nucleation.

### Abp1 interacts dynamically with the sides of actin filaments

**Fig. 2:**
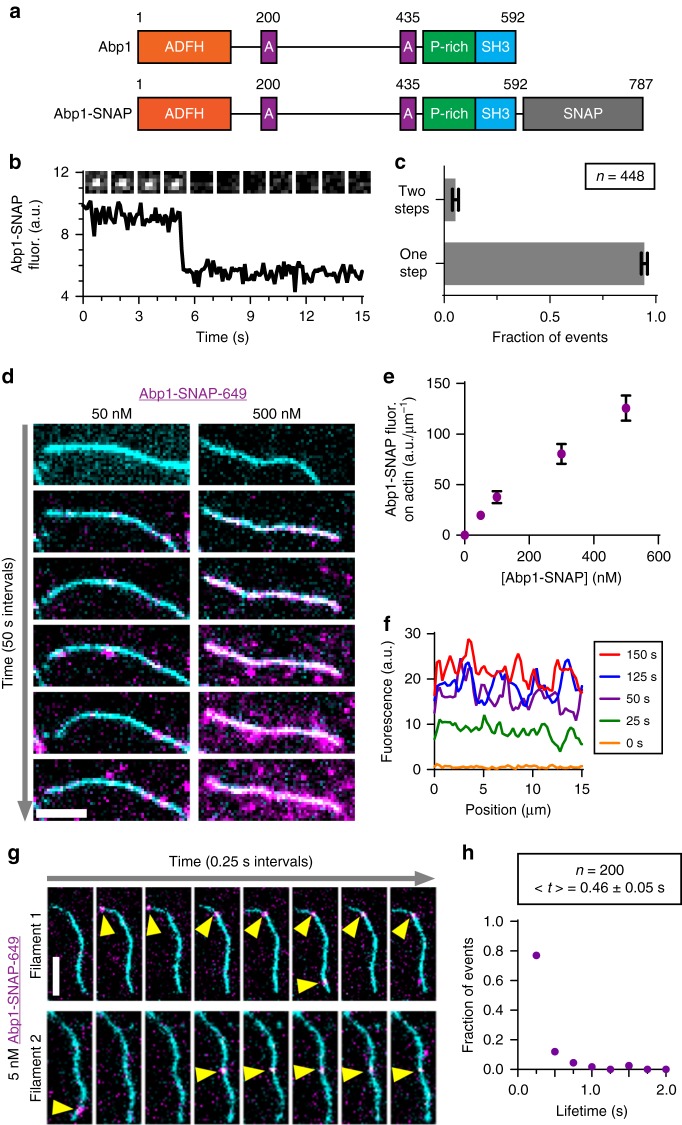
Direct visualization of Abp1 binding to actin filaments. **a** Domain layouts of yeast Abp1 and Abp1-SNAP proteins. **b** Representative step photobleaching of a streptavidin-anchored Abp1-SNAP-biotin-649 molecule. Plot shows fluorescence intensity over time. Inset shows montage of images (1.6 × 1.6 µm) for the same spot analyzed in the plot. **c** Fraction of Abp1-SNAP-biotin-649 molecules (introduced at 10 pM during anchoring) that photobleached in one vs. two steps (>2 photobleaching steps was never observed) from analysis as in (**b**). Mean fractions from 4 separate trials with total of *n* = 448 spots. Error bars, s.e.m. **d** Montage of labeled Abp1-SNAP-649 (magenta) binding to anchored OG-labeled actin filaments (cyan) over time (arrow, 50 s intervals). Scale bar, 5 µm. **e** Concentration-dependent Abp1-SNAP-649 binding to OG-labeled actin filaments as indicated by increasing fluorescence intensity on filaments (normalized to filament length). Data averaged from *n* = 10 filaments per concentration of Abp1-SNAP-649. Error bars, s.d. **f** Distribution of Abp1-SNAP-649 fluorescence on an actin filament at different time points, showing that intensity increases all along the length of the filament over time. **g** Two representative montages (0.25 s intervals) showing dynamic interactions (yellow arrows) of Abp1-SNAP-649 (magenta) molecules on tethered OG-labeled actin filaments (cyan). **h** Distribution of lifetimes of Abp1-SNAP-649 molecules on OG-labeled actin filaments analyzed from *n* = 200 binding events. Average lifetime, <*t*>, with s.e.m.

To further characterize Abp1 role as a type II NPF, we next directly observed Abp1 molecules interacting with actin filaments. To accomplish this, we engineered a C-terminal SNAP-tagged Abp1 and irreversibly labeled it using benzylguanine-dye substrates (Fig. 2a). The SNAP-tag was positioned at the C terminus of Abp1 to avoid interference with its N-terminal actin-binding ADF-homology domain. Abp1-SNAP was labeled with four different dye substrates at >70% labeling efficiency (Surface 549, Surface 649, biotin-DY549, and biotin-DY649), which we used in different experiments below. Dye-labeled Abp1-SNAP showed similar effects to untagged Abp1 in stimulating Arp2/3-mediated branch formation in TIRF assays (Supplementary Figure 2a). Step photobleaching of biotin-anchored Abp1-SNAP spots introduced into TIRF chambers at low concentrations (<1 nM) confirmed that the majority of Abp1 molecules are monomeric (Fig. 2b, c). However, when Abp1-SNAP was introduced at a higher concentration (15 nM) and passively absorbed, about 20% of the spots bleached in two steps (Supplementary Figure  2b, c). This raises the possibility that Abp1 has a modest propensity to form dimers in solution in the absence of other proteins.

To directly observe Abp1 molecules interacting with F-actin, we first assembled and tethered biotin-labeled actin filaments to the glass surface using a biotin-streptavidin-biotin linkage, then flowed in different concentrations of labeled Abp1 and monitored binding over time (Fig. 2d). As expected, binding was concentration dependent (Fig. 2e). Interestingly, the spatial distribution of Abp1 along filaments over time was relatively uniform (Fig. 2f). These observations also showed that Abp1 molecules interacted dynamically with actin filaments, rapidly binding and dissociating. To characterize the lifetimes, we lowered the concentration of labeled Abp1 to 5 nM and increased the frequency of image capture to every 0.25 s. Under these conditions, we observed spots of labeled Abp1 rapidly binding and dissociating (Fig. 2g), with an average dwell time of 0.46 s (Fig. 2h). These properties of Abp1 binding are distinct from ADF/Cofilin, which binds cooperatively and appears on filaments as discrete patches that grow over time^46,47^. Abp1 interacts with F-actin primarily through its ADF-homology domain^18,48^. Therefore, our observations suggest that small differences in otherwise structurally similar ADF-homology domains^40^ can lead to major changes in their interactions with F-actin and their functional effects, as ADF/Cofilin severs and depolymerizes filaments whereas Abp1 does not^18^.

### Abp1 binds to Arp2/3 complex with a 2:1 molar ratio

**Fig. 3:**
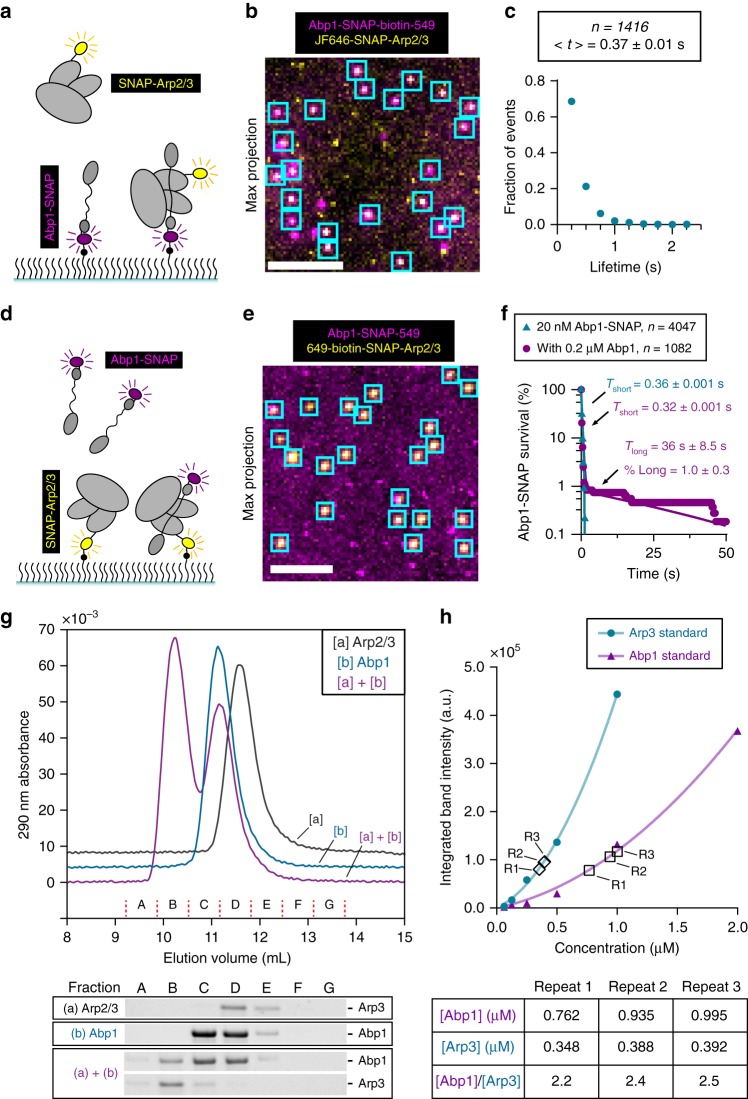
Stoichiometry and dynamics of Abp1 interactions with Arp2/3 complex. **a** Schematic of experimental setup for measuring interactions between labeled Arp2/3 complex and surface tethered labeled Abp1 molecules. **b** Abp1-SNAP-biotin-549 (magenta) molecules were streptavidin-anchored to the surface, then free JF646-SNAP-Arp2/3 molecules (yellow) were flowed in, and binding events were monitored at 0.25 s intervals for 60 s. Image is a representative maximum projection overlay of the two channels, with cyan boxes marking selected sites of colocalization. Scale bar, 5 µm. **c** Lifetime distribution of JF646-SNAP-Arp2/3 molecules binding to anchored Abp1-SNAP-biotin-549 for *n* = 1416 binding events from two trials. Average lifetime, <*t*>, with s.e.m. **d** Schematic of experimental setup with anchored 649-biotin-SNAP-Arp2/3 complex molecules and Abp-SNAP-549 molecules. **e** 649-biotin-SNAP-Arp2/3 complex (*yellow*) was streptavidin-anchored, and then free Abp1-SNAP-549 molecules (magenta) were flowed in, and binding was monitored at 0.25 s intervals for 120 s. Image is a representative maximum projection overlay, as in (**b**). Scale bar, 5 µm. **f** Cumulative survival of Abp1-SNAP-549 molecules bound to anchored 649-biotin-SNAP-Arp2/3 complex spots in the absence (cyan) and presence (magenta) of 0.2 µM unlabeled Abp1. Lifetimes were fit by single or double exponentials as in Fig. 1e, yielding the indicated fit parameters with s.e. from *n* = 10,000 bootstrap samples. **g** Size exclusion chromatography profiles for 0.7 µM Arp2/3 complex [a], 7 µM Abp1 [b], or both mixed. Chromatograms are vertically offset slightly for clarity. Fractions are indicated by capital letters. Below the traces are Arp3 and Abp1 bands from ‘stain-free’ gels of the same fractions (see Supplementary Figure  3a for complete gel). **h** Integrated fluorescence intensity from stain-free gel bands of known amounts of Arp2/3 (circles) or Abp1 (triangles). Data were fit with trendlines (shown) to a second-order polynomial, forced through zero. These curves were used to infer concentration from the integrated intensities of Arp3 and Abp1 bands in the peak fractions of the complex in three technical repeats (R1, R2, R3), marked by hollow diamonds (Arp3) or hollow squares (Abp1). Calculated concentrations and stoichiometry are summarized in the table

Abp1 NPF activity depends not only on its direct interactions with actin filaments, but also on its direct interactions with Arp2/3 complex^18,40^. To examine Abp1 interactions with Arp2/3 complex in the absence of actin, we tethered single Abp1-SNAP-biotin-549 molecules to the glass surface using streptavidin linkage, then flowed in 10 nM JF646-SNAP-Arp2/3 and monitored binding (Fig. 3a), capturing images every 0.25 s. By following the appearance of Arp2/3 complex at sites of immobilized Abp1 (Fig. 3b), we measured the duration of 1416 colocalization events and determined the average dwell time to be 0.37 s (Fig. 3c). This observation suggests that Abp1 monomers interact dynamically with Arp2/3 complex in the absence of actin. The transient nature of these interactions between Abp1 monomers and Arp2/3 complex was somewhat surprising, given that yeast Abp1 remains associated with Arp2/3 complex when purified from cell extracts through multiple chromatography steps^18^.

To address the possibility that anchoring Abp1 monomers might interfere with Arp2/3 complex binding, we altered our experimental design (Fig. 3d), this time anchoring biotin-649-SNAP-Arp2/3 and flowing in 20 nM Abp1-SNAP-549 (Fig. 3e). We measured the duration of 4027 binding events, and determined the average lifetime to be 0.36 s (Fig. 3f, cyan). This value is very similar to the average lifetime for binding observed above when Abp1 was anchored. To further investigate these interactions, we performed another experiment using anchored Arp2/3 complex, except we flowed in a higher concentration of Abp1 (220 nM total, consisting of 20 nM Abp1-SNAP-549 and 200 nM unlabeled Abp1). These conditions were used to avoid background problems caused by higher concentrations of labeled protein. At this higher concentration of Abp1, we now observed long-lived interactions between labeled Abp1 and Arp2/3 complex, lasting 36 ± 8.5 s (mean ± s.e., determined from *n* = 1082 events) (Fig. 3f, magenta). These observations raise the possibility that at the higher concentration of 220 nM, more than one molecule of Abp1 binds to Arp2/3 complex, leading to longer-lived binding events.

To more directly address the stability and stoichiometry of Abp1 binding to Arp2/3 complex in solution, we performed size exclusion chromatography, and quantified the protein concentrations in the resulting peaks. Abp1 alone and Arp2/3 complex alone each migrated as a single peak. Adding an excess of Abp1 (7 μM) to Arp2/3 complex (0.7 μM) gave rise to a new peak that eluted earlier than either Abp1 or Arp2/3 alone (Fig. 3g and Supplementary Figure 3a, b). Analysis of the larger complex by modified tryptophan fluorescence intensity in ‘stain-free’ gels (Fig. 3g, fraction B) confirmed that it contains both Abp1 and Arp2/3 complex and allowed us to quantify their levels. Using a standard curve of known concentrations of each protein, we determined that the molar ratio of Abp1 to Arp2/3 complex is ~2:1 (Fig. 3h and Supplementary Figure 3c). These results indicate that each Arp2/3 complex has multiple Abp1 molecules bound, similar to what has been shown for two other Arp2/3 ligands, WASP and GMF^12,42^.

### Abp1 stably associates with actin filament branch junctions

**Fig. 4:**
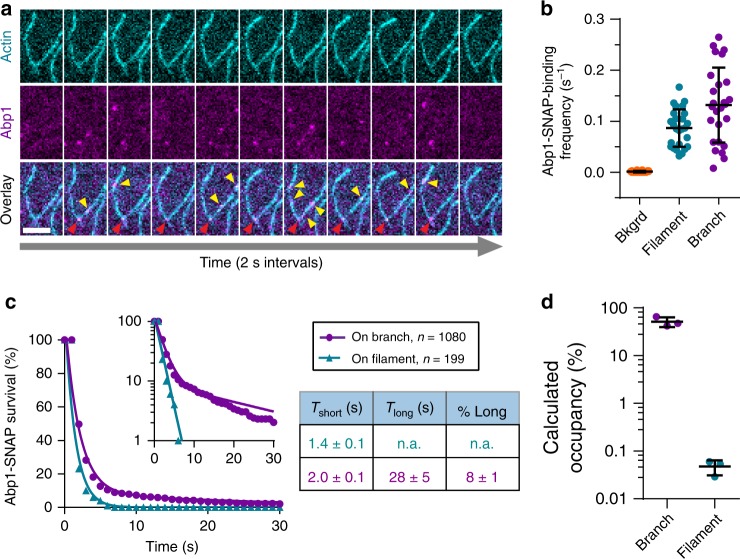
Abp1 associates more stably with branch junctions than actin filaments. **a** Example montage showing Abp1-SNAP-649 molecules (magenta) interacting with actin filament sides (yellow arrows) and branch junctions (red arrows). Reactions contain 1 µM actin (10% OG-labeled) with 10 nM Abp1-SNAP-649 and 8 nM Arp2/3 complex. Montage shows 2 s intervals. Scale bar, 5 µm. **b** Abp1-SNAP-649 binding frequencies to background, actin filament sides, and filament branch junctions, expressed as the average number of Abp1-SNAP binding events within a 1.14 µm^2^ detection box at each location. Values measured from *n* = 26 binding targets for each condition from the same recordings as in (**a**). Error bars, s.d. **c** Cumulative survival distributions (circles and triangles) of Abp1-SNAP molecules binding to actin filament sides vs. branch junctions as in (**e**), for *n* = 1080 binding events at branch sites and *n* = 199 binding events at filament sides from three trials. Measured lifetimes for Abp1-SNAP binding to filament sides and branch sites were fitted to single or double exponential distribution (lines), respectively, yielding the indicated fit parameters ± s.e. **d** Calculated Abp1 binding occupancy (as a percentage) of available sites on actin filament sides vs. branch junctions. Data are from *n* = 3 separate trials. Error bars, s.d.

Having separately examined binding of Abp1 to F-actin and Arp2/3 complex, we next examined Abp1 interactions with preformed branched actin filaments, where both F-actin and Arp2/3 complex are present. To accomplish this, we assembled actin filaments in the presence of Arp2/3 complex and 10 nM Abp1-SNAP-649 (Fig. 4a), capturing images at 1 s intervals to reduce photobleaching. Abp1-SNAP-649 binding to background surface locations without actin (1.14 µm^2^ detection box) was very low compared to binding to filament sides and branch junctions (Fig. 4b). The distribution of branch colocalization events (*n* = 1080) included at least two distinct components, one that is short-lived (2.0 ± 0.1 s) and one that is much longer-lived (28 ± 5 s) (Fig. 4c). Interestingly, the short-lived component of the detected branch colocalization events has a similar lifetime to Abp1-SNAP binding to filament sides (1.4 ± 0.1 s, *n* = 199 events). This raises the possibility that the short-lived binding events observed within the branch detection box actually represent binding of Abp1 to the filament lattice, rather than the branch junction. Note that Abp1-SNAP lifetime on filaments was determined to be 0.46 s in the earlier experiments (Fig. 2g), from recordings at 0.25 s intervals (Fig. 2h). The recordings here were made at 1 s intervals, and therefore miss some short-lived binding events and overestimate the average duration. Regardless, the remaining 10% (long-lived) observed binding events for Abp1-SNAP-649 are likely to represent branch junction binding, and have an average lifetime of 27.5 s. These observations suggest that Abp1 associates much more stably with branch sites than filament sides. Further, this is consistent with a higher Abp1 occupancy of branch sites comparing to filament sides, with a calculated 60% occupancy by labeled Abp1 at available branch sites (assuming 1 binding site per branch) vs. <0.1% occupancy of available binding sites along filament sides (assuming 370 binding sites per µm F-actin). Thus, Abp1 likely binds nearly three orders of magnitude more tightly to branches than to filament sides (Fig. 4d).

### Abp1 protects branch junctions from GMF-induced dissociation

**Fig. 5:**
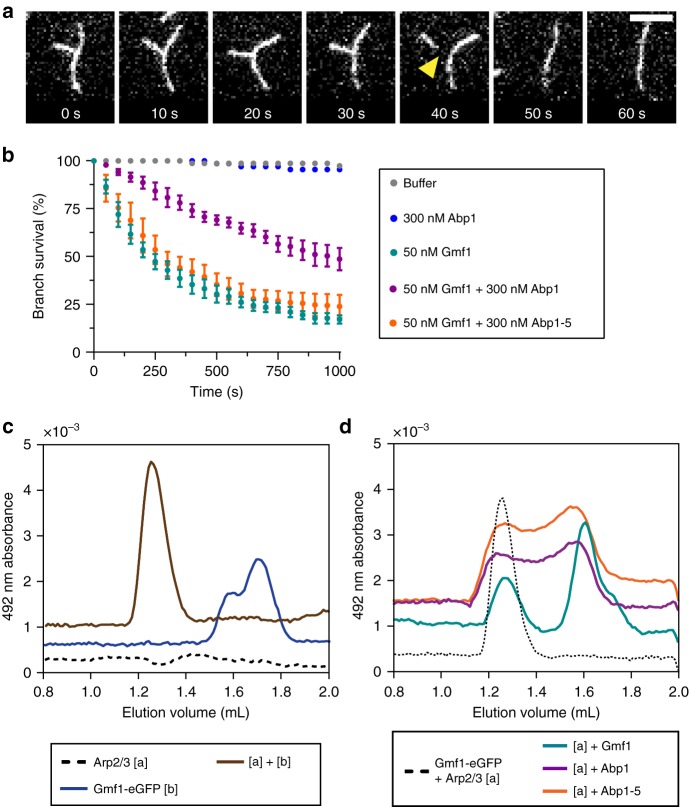
Abp1 protects actin filament branches from GMF-induced debranching. **a** Time points from a TIRF microscopy debranching assay, with yellow arrow marking debranching event. Branched filaments were first polymerized and biotin-anchored in the TIRF chamber with actin (10% OG-labeled, 0.5% biotin-labeled), Arp2/3 complex, and GST-VCA. Then, proteins of interest were flowed in, and effects on branch stability were monitored over time. Scale bar, 5 µm. **b** Cumulative survival curves of branches in the presence of different proteins, quantified from *n* = 50 branches in each of four independent trials per condition. For the two negative controls (buffer alone, and 300 nM Abp1), data are from a single trial. Error bars, s.e.m. **c** Size exclusion chromatography profiles for the migration of 0.8 µM Gmf1-eGFP, monitored by absorbance at 492 nm, in the presence (brown line) and absence (blue line) of 1 µM Arp2/3 complex. The dotted line shows that Arp2/3 complex alone (1 µM) has no appreciable signal at 492 nm. **d** Size exclusion chromatography competition assay for Arp2/3 complex binding, following the absorbance of the Gmf1-eGFP absorbance at 492 nm. The dotted line shows the migration of Gmf1-eGFP (0.8 µM) in the presence of Arp2/3 complex (1 µM), and the colored lines show how the migration of Gmf1-eGFP with Arp2/3 complex is affected by the further addition of binding competitors: 5 µM unlabeled Gmf1 (cyan line), 1 µM Abp1 (magenta line), and 1 µM Abp1-5 (orange line). Chromatograms are vertically offset for clarity

GMF is another ADF-homology domain protein that directly binds Arp2/3 complex and targets branch junctions; however, its function appears to be very different from that of Abp1, as it inhibits Arp2/3-dependent nucleation and catalyzes filament debranching^41^. Given that GMF and Abp1 both have ADF-homology domains and target Arp2/3 complex at branch junctions, we asked how Abp1 might affect GMF-induced debranching. To address this, we developed an in vitro debranching assay, in which we first assembled branched and sparsely anchored filaments in TIRF chambers, then flowed in GMF and/or Abp1 and monitored debranching events over time, producing cumulative branch survival curves (Fig. 5a, b). As expected, branches remained stable after flowing in control buffer or 300 nM Abp1 alone, but were rapidly lost after flowing in yeast GMF (Gmf1) (Fig. 5b). The debranching activity we observed for GMF were similar to what we previously observed in assays performed under actin assembly conditions^41^. However, the use here of preformed, branched, and anchored actin filaments allowed us to more accurately monitor branch stability over longer periods of time, and improve quantification of debranching events. The addition of Abp1 slows the overall rate of debranching by GMF (Fig. 5b). Thus, Abp1 protects branches from GMF debranching. In contrast, Abp1-5 failed to attenuate Gmf1-mediated debranching, which suggests that the surface on the ADF-homology domain mutated in Abp1-5 (equivalent to the Cof1-22 site on ADF/Cofilin^49^) is required not only for productive interactions between Abp1 and Arp2/3 leading to nucleation, but also blocking GMF effects (see Discussion).

One simple explanation for the observations above is that Abp1 and GMF compete for binding Arp2/3 complex. To test this model, we used a gel-filtration-based competition assay that monitors Gmf1-eGFP migration^42^. Gmf1-eGFP alone (0.8 µM) eluted from the size exclusion column at 1.7 mL, but in the presence of Arp2/3 complex (1 µM), Gmf1-eGFP eluted at 1.3 mL, consistent with binding of Gmf1-eGFP to Arp2/3 complex (Fig. 5c). Addition of excess unlabeled Gmf1 (5 µM) largely reversed this shift, returning Gmf1-eGFP elution to 1.7 mL, as expected for competitive binding to Arp2/3 complex (Fig. 5d). Similar effects were observed if instead Abp1 (1 µM) was added (Fig. 5d), suggesting that Abp1 competes with Gmf1-eGFP for Arp2/3 binding. Surprisingly, the ADFH mutant, Abp1-5, which fails to protect branch junctions from GMF-mediated debranching, was able to compete with Gmf1-eGFP for Arp2/3 complex binding similarly to wild type Abp1 (Fig. 5d) (see Discussion).

### Structures of Abp1-bound Arp2/3 complex

**Fig. 6:**
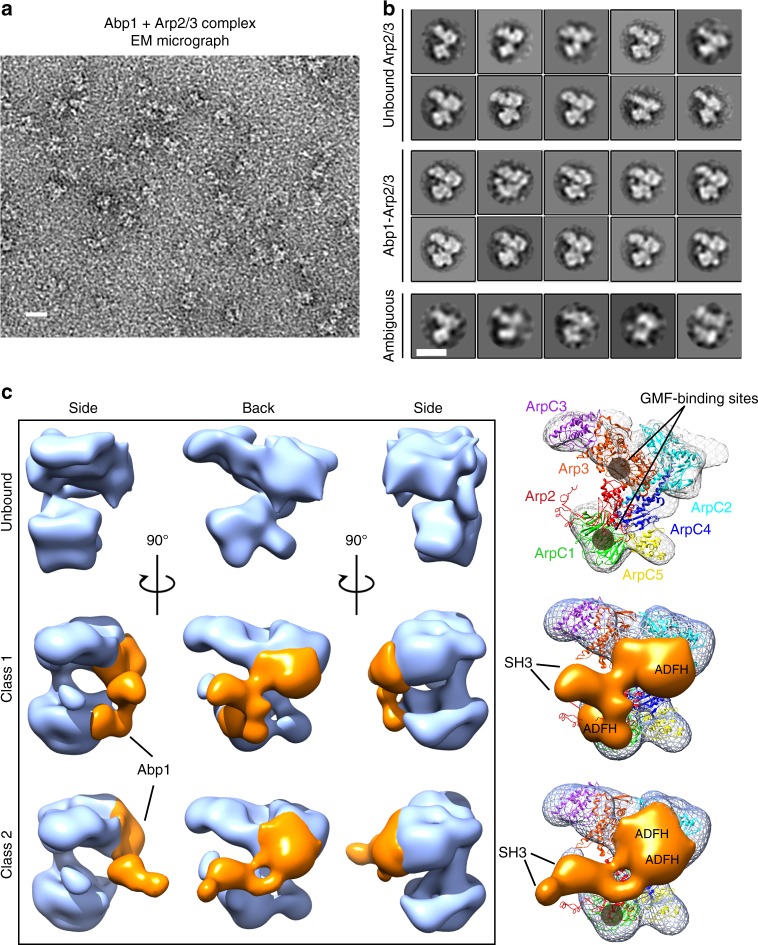
Two structures of Abp1-bound Arp2/3 complex. **a** Representative raw micrograph of negatively stained Abp1 and Arp2/3 complex imaged by transmission electron microscopy. Scale bar, 20 nm. **b** All 2D class averages generated from the raw images, which fell into three broad categories: unbound Arp2/3 complex, Abp1-bound Arp2/3 complex, and ambiguous (excluded from 3D reconstructions). Scale bar, 10 nm. **c** 3D reconstructions of unbound Arp2/3 complex and two different conformations of Abp1-bound Arp2/3 complex (Class 1 and Class 2). In the two Abp1-bound reconstructions, extra mass from Abp1 (orange) is shown bound to Arp2/3 complex (light blue). Right column shows overlay of Abp1 density (orange) on Arp2/3 complex (meshed surface) docked with crystal structure of Arp2/3 complex. Gray circles indicate sites of GMF binding on Arp2/3 complex. Assignments of Abp1 domains are based on relative sizes of the extra masses, and data in this study showing the importance of the ADFH domains in Abp1 competing with GMF for Arp2/3 complex binding

Finally, to gain structural insights into Abp1 effects on Arp2/3 complex, we used electron microscopy (EM) and single-particle analysis to determine the structure of Abp1-bound Arp2/3 complex. Abp1 and Arp2/3 complex were mixed at a 1:1 molar ratio and absorbed to grids, negatively stained, and then imaged by transmission electron microscopy (Fig. 6a). From a total of 4328 untilted views, 25 different two-dimensional (2D) class averages were generated (Fig. 6b). Using random conical tilt method, we generated 3D reconstructions. All particles (total of 6264, tilted and untilted) fell into one of three categories: free Arp2/3 complex (56% of particles), and two distinct Abp1-bound Arp2/3 complexes, which we refer to as Class 1 (30% of particles) and Class 2 (14% of particles) (Fig. 6c). In each Abp1-bound Arp2/3 structure, the new density suggests that Abp1 forms a dimer when it is bound to Arp2/3 (Fig. 6c), which agrees with our gel-filtration and stoichiometry analysis above (Fig. 3h).

In each of the Abp–Arp2/3 structures, the larger masses in the Abp1 molecules likely correspond to the globular N-terminal ADFH domains, and the smaller masses at the other ends of the molecules likely correspond to the C-terminal SH3 domains (Fig. 6c). In the Class 1 structure, Abp1 dimers show a ‘split’ appearance, with the large mass of one Abp1 molecule binding near the Arp3 subunit and the large mass of the other molecule binding near the p40/ARPC1 and Arp2 subunits (Fig. 6c). Interestingly, these are the same two sites on Arp2/3 complex proposed to bind GMF (Fig. 6c)^42,43,50,51^. Thus, the Class 1 structure of Abp1–Arp2/3 provides structural support for our model that the ADFH domain of Abp1 competes with GMF for Arp2/3 binding to protect branches from GMF-induced debranching. In addition, another type II NPF, Cortactin, binds to similar subunits on Arp2/3 complex^23,29^, which raises the possibility of a related mechanism to Abp1.

In the Class 2 structure, the Abp1 molecules are aligned in parallel in the dimer, with one molecule binding near Arp3 (as observed in the Class 1 structure), and the other not contacting the Arp2/3 complex (Fig. 6c). This configuration has implications for the mechanism by which Abp1 recruits and stabilizes Arp2/3 complex on filament sides, suggesting that the ADF-homology domain of one Abp1 molecule in the dimer may be used to bind Arp2/3 complex while the ADF-homology domain of the other molecule is available to bind F-actin. In both structures, it appears that Abp1 dimerization is mediated by its C-terminal half, possibly resulting from intermolecular interactions between the SH3 domain and adjacent polyproline sequences.

## Discussion

Abp1 and Cortactin were classified as type II NPFs almost two decades ago^18–20^, and yet there has been limited progress in defining their roles and mechanisms in controlling Arp2/3 complex-mediated actin assembly. To close this gap, we used single-molecule TIRF microscopy to define in real time Abp1 effects on Arp2/3-mediated actin branch formation and turnover. Further, we used single-particle EM to examine the structural basis of Abp1 interactions with Arp2/3 complex. Our single-molecule TIRF analysis revealed that Abp1 strongly enhances Arp2/3-mediated daughter branch nucleation, and that it does so by stabilizing Arp2/3 complex on the sides of mother filaments, which is a rate-limiting step in branched nucleation^17^. These data provide the first direct observation of any NPF stabilizing Arp2/3 complex interactions with actin filament sides and may be mechanistically informative for understanding other type II NPFs. We also observed that Abp1 can work in combination with dimerized VCA domain of WASP/Las17, a type I NPF, to enhance Arp2/3-mediated actin nucleation. Synergy between Cortactin and WASP has been observed in a previous study, which is due at least in part to the ability of Cortactin to accelerate the release of VCA from the barbed ends of daughter filaments^27^. Thus, in the future it will be important to determine whether Abp1 has similar mechanistic capabilities and, reciprocally, whether Cortactin can stabilize Arp2/3 complex on filament sides as we have shown here for Abp1.

In addition, we uncovered a new function for Abp1 in protecting branches from GMF-induced dissociation. This antagonistic relationship between Abp1 with GMF, two ADFH domain proteins, in regulating Arp2/3 complex may be used to balance branch stability and turnover in cells. Note that, this function would not be predicted for Cortactin, which lacks an ADF-homology domain, and therefore may be unique to Abp1. This function of Abp1 is also consistent with our EM structures, which show that the two ADF-homology domains of the Abp1 dimer contact surfaces on Arp2/3 complex overlapping with the proposed GMF-binding sites^42,43,50^. Further, the antagonistic relationship may extend to the control of actin nucleation by Arp2/3 complex, as GMF binds free Arp2/3 complex to block VCA-stimulated actin nucleation^41,52^. Thus, Abp1 may oppose these inhibitory effects of GMF to promote actin nucleation. Unexpectedly, we observed that the Abp1-5 mutant, which fails to protect branches from GMF-mediated debranching, still competes with GMF for Arp2/3 complex binding in solution. One possibility is that the ADFH domain surface mutated in Abp1-5 is required to regulate Arp2/3 complex specifically in the context of a branch junction. Indeed, Arp2/3 complex at filament branch sites has a distinct conformation from Arp2/3 complex in solution^53^. It is worth noting that the ADFH domain of GMF has two separate functional surfaces, site 1 and site 2, with site 2 regulating branch-bound Arp2/3 complex but not free Arp2/3 in solution^42^. The Abp1-5 mutation targets the analogous surface to the *gmf-17* ‘site 2’ mutation, and in both cases (GMF and Abp1), this site is used specifically to regulate Arp2/3 complex incorporated into F-actin branches, and not free Arp2/3 complex in solution.

These findings also offer insights into a long-standing dilemma over the conservation of Abp1 function in Arp2/3 regulation. While yeast Abp1 has NPF activity, which depends on its two acidic motifs^18^, mammalian Abp1 alone shows no NPF activity and lacks the acidic motifs^48,54^. Nonetheless, mammalian Abp1 has been shown to regulate Arp2/3-dependent functions in vivo^36,37,39,48^, and binds directly to WASP using its SH3 domain, stimulating release of WASP from autoinhibition to promote nucleation^37^. Our observations add to this understanding, suggesting that mammalian Abp1 may promote recruitment of WASP-Arp2/3 complexes to filament sides to enhance nucleation. Moreover, the ADF-homology domain is conserved between yeast and mammalian Abp1, and therefore may be used in both cases to antagonize GMF, and thus stabilize branches. Why yeast Abp1 has evolved to have acidic motifs may be related to the absence of Cortactin in yeast. Like yeast Abp1, Cortactin has acidic motifs, and shares partially overlapping mechanistic effects on Arp2/3 complex with mammalian Abp1. Thus, type II NPF duties may be split between mammalian Cortactin and Abp1, with each performing specialized Arp2/3 regulatory functions, whereas in yeast these functions are combined in one protein, Abp1.

Finally, our two EM structures provide important mechanistic insights into how Abp1 promotes nucleation and stabilizes branches. Our data show that Abp1 alone in solution is mostly monomeric, but that it binds to Arp2/3 complex with a stoichiometry of 2:1, as indicated by our size exclusion chromatography analysis and our two distinct 3D reconstructions of Abp1-bound Arp2/3 complex. In both structures, Abp1 dimerizes when it is bound to Arp2/3 complex, and one of the two ADF-homology domains in the dimer is bound near the Arp3 subunit. However, the other ADF-homology domain is found in two distinct locations in the two structures, which may represent snapshots of Abp1 performing its two different functions. In the Class 2 structure, the other ADF-homology domain appears available for F-actin binding, which would enable Abp1 to recruit Arp2/3 complex to filament sides and promote nucleation, as suggested by our single-molecule observations. In the Class 1 structure, the other ADF-homology domain is instead bound to a second site on Arp2/3, near the Arp2 and p40/ARPC1 subunits, and therefore is predicted to more effectively block GMF effects. Thus, the ADF-homology domain of Abp1 is versatile, interacting with both F-actin and Arp2/3 complex, whereas some other members of the ADF-homology family bind specifically to actin (e.g., ADF/Cofilin and Coactosin) or Arp2/3 complex (e.g., GMF). These observations establish two separate mechanistic roles for Abp1 in regulating Arp2/3 complex, one in promoting actin nucleation and the other in stabilizing branches against GMF. Collectively, this work broadens our understanding of how type II NPFs contribute to the coordinated regulation of Arp2/3 complex-mediated actin assembly and turnover.

## Methods

### Reagents and plasmids

All materials were obtained from Sigma-Aldrich (St. Louis, MO) unless noted. Expression plasmid pGEX-Abp1 and pGEX-Abp1-5 were generated by PCR amplifying wild-type and mutant *S. cerevisiae* Abp1 genes from plasmids previously generated^40^ and subcloning the resulting inserts into *Bam*HI and *Not*I sites of pGEX-6P1. Expression plasmid pGEX-Abp1-SNAP was generated by subcloning the *S. cerevisiae* Abp1 gene without the STOP codon into the *Bam*HI and *Eco*RI sites of pGEX-6P1, then subcloning the SNAP-tag sequences into *Eco*RI and *Not*I sites. All constructs were verified by sequencing.

### Protein expression and purification

Rabbit skeletal muscle actin^55^ was purified from acetone powder generated from frozen ground hind leg muscle tissue of young rabbits (PelFreez, Rogers, AR). Lyophilized acetone powder stored at −80 °C was mechanically sheared in a coffee grinder, resuspended in G-buffer (5 mM Tris-HCl pH 7.5, 0.5 mM dithiothreitol (DTT), 0.2 mM ATP, 0.1 mM CaCl_2_), and then cleared by centrifugation for 20 min at 50,000 × *g*. Actin was polymerized by the addition of 2 mM MgCl_2_ and 50 mM NaCl and incubated overnight at 4 °C. F-actin was pelleted by centrifugation for 150 min at 361,000 × *g*, and the pellet solubilized by dounce homogenization and dialyzed against G-buffer for 48 h at 4 °C. Monomeric actin was then precleared at 435,000 × *g*, and loaded onto a S200 (16/60) gel-filtration column (GE Healthcare, Marlborough, MA) equilibrated in G-Buffer. Fractions containing actin were stored at 4 °C. For labeling actin with biotin or Oregon Green (OG), the F-actin pellet described above was dounced and dialyzed against G-buffer lacking DTT. Monomeric actin was then polymerized by adding an equal volume of 2× labeling buffer (50 mM Imidazole pH 7.5, 200 mM KCl, 0.3 mM ATP, 4 mM MgCl_2_). After 5 min, the actin was mixed with a fivefold molar excess of NHS-XX-Biotin (Merck KGaA, Darmstadt, Germany) or OG-488 iodoacetamide (Invitrogen, Carlsbad, CA) resuspended in anhydrous dimethylformamide, and incubated in the dark for 15 h at 4 °C. Labeled F-actin was pelleted as above, and the pellet was rinsed briefly with G-buffer, then depolymerized by dounce homogenization, and dialyzed against G-buffer for 48 h at 4 °C. Labeled, monomeric actin was purified further on an S200 (16/60) gel-filtration column as above. Aliquots of biotin-conjugated actin were snap frozen in liquid nitrogen and stored at −80 °C. OG-488-actin was dialyzed for 15 h against G-buffer with 50% glycerol and stored at −20 °C.

Untagged yeast Arp2/3 complex was purified from commercially sourced, fresh RedStar yeast cakes as previously described^56^. Yeast cells were resuspended, washed, and centrifuged three times in 10 mM Imidazole pH 7.0 plus 150 mM NaCl. Cells were then resuspended in 2 mL of Buffer UB/g wet cell pellet weight (Buffer UB: 50 mM HEPES pH 7.5, 100 mM KCl, 1 mM EGTA, 3 mM MgCl_2_, 1 mM DTT, 2 µg/mL antipain and 20 µg/mL leupeptin). Phenylmethylsulfonyl fluoride (PMSF; 1 mM) was added part way through resuspending the cell pellet. Resuspended cells were flash frozen by dripping them directly into liquid nitrogen, and then stored at −80 °C. To purify Arp2/3 complex, ~800 g of frozen cell suspension was thawed, lysed by extrusion using a M-110P extruder (Microfluidics Corp, Westwood, MA) operated at 22,000 psi. Cells were clarified by centrifugation at 138,000 × *g* at 4 °C for 1 h. Supernatants were collected and filtered through cheesecloth and brought to 25% saturation of ammonium sulfate at 4 °C, and centrifuged. The supernatant was collected and brought to 55% saturation in ammonium sulfate, and the mixture was centrifuged. The pellet was retained and resuspended in Buffer A (20 mM HEPES pH 7.5, 25 mM KCl, 1 mM MgCl_2_, 1 mM DTT, 0.5 mM EGTA, 0.1 mM ATP), and set to dialyze against Buffer A overnight using 50 kDa molecular weight cutoff dialysis tubing. Dialysate was centrifuged at 44,000 × *g* in a JA25.5 rotor (Beckman Coulter Inc., Brea, CA) for 30 min, then set aside on ice while a VCA column was prepared using N-WASP GST-VCA expressed in BL21(DE3) T1^R^ *Escherichia coli*. GST-VCA-expressing *E. coli* were lysed by extrusion, clarified, enriched using DEAE (diethylaminoethanol) sepharose ion exchange chromatography, then applied to Glutathione sepharose beads (GE Healthcare). The GST-VCA beads were washed extensively, equilibrated in Buffer A, and resuspended in ~1.5 column volumes of Buffer A. These beads were mixed with the reserved dialysate, and allowed to bind in batch mode with rocking at 4 °C for 25 min, and then beads were collected in a 2.5 cm low pressure chromatography column. The unbound flow-through fraction was retained. Beads were washed with Buffer A, then with Buffer B (20 mM HEPES pH 7.5, 200 mM KCl, 1 mM MgCl_2_, 1 mM DTT, 0.5 mM EGTA, 0.1 mM ATP). Finally, Arp2/3 complex was eluted from the beads using six sequential 0.7 column volume applications of Buffer C (20 mM HEPES pH 7.5, 200 mM MgCl_2_, 25 mM KCl, 1 mM DTT, 0.5 mM EGTA, 0.1 mM ATP). Following elution, GST-VCA beads were again equilibrated in Buffer A and mixed with the retained flow-through fraction, and the process repeated. Combined VCA column eluate fractions were exchanged into Buffer QC (20 mM Tris-HCl pH 8, 1 mM DTT, 10 mM EGTA, 2 mM MgCl_2_) using a 50 mL Sephadex G-25 medium desalting column and applied to a 4 mL SOURCE15Q ion exchange column. SOURCE15Q column was equilibrated in Buffer QC, and operated with Buffer QC and Buffer QD (20 mM Tris-HCl pH 8, 1 mM DTT, 10 mM EGTA, 2 mM MgCl_2_, 1 M NaCl). Column was washed with 4% Buffer QD, and Arp2/3 complex was eluted using a linear gradient from 4% Buffer QD to 29% Buffer QD developed over 50 column volumes, with fractionation. Relevant fractions were assessed by sodium dodecyl sulfate–polyacrylamide gel electrophoresis (SDS-PAGE), and Arp2/3 complex containing fractions were pooled. At this point, the most prominent contaminant appears to be endogenous Abp1^57^. To remove contaminants, Arp2/3 complex was applied to Superdex 200 pg 26/600 column, run using a buffer of 100 mM KCl, 10 mM HEPES, pH 7.5, 1 mM MgCl_2_, 1 mM EGTA, 0.5 mM DTT, 100 µM ATP. Peak fractions were assessed by SDS-PAGE, selecting for fractions rich in Arp2/3 complex but depleted of Abp1, and then reapplied to the Superdex 200 column for a final round of polishing purification in the same buffer. Peak fractions were assessed using SDS-PAGE, concentrated, supplemented with one half-volume of 100 mM KCl, 10 mM HEPES pH 7.5, 1 mM MgCl_2_, 1 mM EGTA, 0.5 mM DTT, 0.1 mM ATP, and 60% w/v sucrose (to a final concentration of 20% w/v sucrose), and snap frozen in liquid nitrogen.

3HA-TEV-SNAP-tagged *S. cerevisiae* Arp2/3 complex was purified from a previously engineered yeast strain, BGY1430^17^. Briefly, BGY1430 cells were grown in YPD media until *A*_600_ = 1–2, washed, resuspended in a 2:1 w/v ratio with HEK buffer (20 mM Na^+^-HEPES pH 7.5, 1 mM EDTA, 50 mM KCl), and snap frozen into droplets in liquid nitrogen. Cells were mechanically sheered in a coffee grinder using liquid nitrogen, then thawed by addition of HEK buffer plus protease inhibitors: 1 mM PMSF and 0.5 μg/mL each of antipain, chymostatin, aprotinin, pepstatin A, and leupeptin. The lysate was cleared by centrifugation at 300,000 × *g* for 20 min, and the supernatant was passed three times through a homemade 1 mL anti-HA antibody column. To generate this column, 1 mL of cyanogen bromide-activated agarose beads (GE Healthcare) was mixed with 6 mg of HA monoclonal antibody (Clone HA-7, #H9658, Sigma). The column was washed three times with HEK buffer plus 1 mM ATP (HEK_ATP_) and three times with HEK_500_ buffer (20 mM HEPES, pH 7.5, 1 mM EDTA, 500 mM KCl) plus 1 mM ATP. The column was equilibrated with HEK_ATP_ buffer, and then loaded with 10 µM of BG-JF646-SNAP-dye substrate^58^ to label the bound Arp2/3 complex for 2 h at 25 °C in the dark. Excess SNAP-dye was washed away with HEK_ATP_ buffer, and SNAP-Arp2/3 complex (lacking 3HA tag) was released from the column by TEV protease digestion, and aliquots were snap frozen in liquid nitrogen and stored at −80 °C.

Full-length Abp1 polypeptides (GST- and GST-SNAP-tagged) were expressed in *E. coli* strain BL21(DE3) pLysS. Cells were grown in TB media to OD_600 _= 1.0 at 37 °C, then protein expression was induced for 16 h at 18 °C by addition of 0.8 mM isopropyl-β-D-thiogalactopyranoside (IPTG). Cells were harvested by centrifugation and resuspended in 50 mL of 2 × phosphate-buffered saline (PBS), supplemented freshly with 1 mM EDTA, 1 mM DTT, 1 mM PMSF, and a standard mixture of protease inhibitors. Cells were lysed by sonication on ice and clarified by centrifugation at 14,000 × *g* for 20 min. The resulting supernatant was incubated with 1 mL glutathione-agarose beads for at least 2 h at 4 °C, rotating. Beads were washed five times with 2× PBS before Abp1 was released by cleavage with PreScission protease (GE Healthcare) for 16 h at 4 °C. Released Abp1 was purified further on a MonoQ (5/5) anion exchange column (GE Healthcare) equilibrated in HEK buffer (20 mM Na^+^-HEPES pH 7.5, 1 mM EDTA, 50 mM KCl), and eluted with a 20 column volume linear salt gradient (0–1 M KCl). Peak fractions were determined by SDS-PAGE analysis and concentrated. For purifying SNAP-tagged Abp1 (Abp1-SNAP), the same procedure was followed except the concentrated fractions from the MonoQ column were supplemented with 1 mM DTT and incubated at 25 °C for 2 h with a 1–5 molar excess of SNAP-Surface dye (New England Biolab, Ipswich, MA). Synthesis of biotin-conjugated SNAP-Surface dyes, BG-549-PEG-biotin, and BG-649-PEG-biotin have been described previously^28,59^. Labeling efficiency was determined spectrophotometrically using absorbance at 650 nm and an extinction coefficient of 250,000 M^−1^cm^−1^ for Surface 649 and BG-649-PEG-biotin, or absorbance at 550 nm and an extinction coefficient of 150,000 M^−1^ cm^−1^ for Surface 549 and BG-549-PEG-biotin. Dye absorbance was combined with protein absorbance at 280 nm with an extinction coefficient of 81,360 M^−1^ cm^−1^ for Abp1-SNAP to yield the labeling efficiency. The final protein product was exchanged into HEKDG_5_ (HEK with 1 mM DTT and 5% glycerol) using a PD-10 desalting column (GE Healthcare), aliquoted, snap frozen in liquid N_2_, and stored at −80 °C.

*S. cerevisiae* Gmf1 and Gmf1-eGFP were expressed as cleavable glutathione *S*-transferase (GST) fusions in *E. coli* strain BL21(DE3) T1^R^. Gmf1-eGFP is an internal fusion of eGFP with a flexible linker in Gmf1, positioned between amino acid residues 82 and 83^42^. Cells were grown to mid log phase at 37 °C and expression was induced for 16 h at 20 °C by addition of 1 mM IPTG. Cells were harvested by centrifugation, and each 1 L of culture was resuspended in 30 mL of Buffer GSH-WB1 (200 mM NaCl, 20 mM Tris/HCl pH 8.0, 2 mM EDTA, 2 mM DTT). During resuspension, 1 mM PMSF was added. Cells were lysed by extrusion and clarified by centrifugation at 46,000 × *g* in a JA25.50 rotor (Beckman Coulter Inc.) for 30 min at 4 °C. Supernatants were mixed with glutathione Sepharose 4B beads (GE Healthcare) and rocked at 4 °C for 40 min, then collected in a 1.2 cm wide disposable column. Beads were washed three times with 10 column volumes of Buffer GSH-WB1. GST tags were removed by overnight, in-column digestion at 4 °C with HRV3C protease (Novagen Inc., Madison, WI). Released Gmf1 and Gmf1-eGFP were collected, and loaded on a homemade 4 mL SOURCE15Q anion exchange column. The column was run with QA7I buffer (10 mM Imidazole pH 7.0, 1 mM DTT) and QB7I buffer (10 mM Imidazole pH 7.0, 1 mM DTT, 1 M NaCl). The column was equilibrated at 3% QB7I. The collected protein sample was diluted fourfold with QA7I and applied to the column, and then proteins were eluted with a 25 column volume linear gradient of CB71 (10–60%). Peak fractions containing Gmf1 or Gmf1-eGFP were collected and flash frozen in 0.6 mL aliquots. Prior to use, an aliquot was thawed, centrifuged for 10 min at 15,000 × *g* at 4 °C, and the upper 0.5 mL was applied to a Superdex 200 (10/300) column (GE Healthcare) equilibrated in 10 mM Na^+^-HEPES pH 7.5, 50 mM KCl, 1 mM EGTA pH 8.0, 2 mM MgCl_2_, 0.5 mM DTT, and 0.1 mM ATP. The concentration of Gmf1 was assessed by absorbance at 280 nm, and the concentration of Gmf1-eGFP by absorbance at 496 nm. Abp1 protein used to test competition with Gmf1 in binding Arp2/3 complex was purified by the same protocol as above for Gmf1. By the SOURCE15Q step, only one dominant Abp1 containing peak was observed. This peak was collected, aliquoted and frozen, and applied to the Superdex 200 10/300 gel-filtration column as described for Gmf1 above.

### Total internal reflection fluorescence microscopy

In all experiments, 24 × 60 mm coverslips (Fisher Scientific, Pittsburgh, PA) were first cleaned by sonication in detergent for 60 min, followed by successive sonication in 1 M KOH and 1 M HCl for 20 min each, then sonication in ethanol for 60 min. Coverslips were next washed extensively with H_2_O, dried in an N_2_-stream, layered with 200 µL of 80% ethanol pH 2.0, 2 mg/mL methoxy-poly(ethylene glycol)-silane molecular weight (M.W.) 2000 and 2 µg/mL biotin-poly(ethylene glycol)-silane M.W. 3400 (Laysan Bio Inc., Arab, AL), and incubated for 16 h at 70 °C. Flow cells were assembled by rinsing the coated coverslips extensively with H_2_O, then attaching it to a plastic flow chamber (Ibidi, Martinsried, Germany) with 2.5 cm × 2 mm × 120 µm double-sided tape (Grace Bio-Labs, Bend, OR) and epoxy resin (Devcon, Riviera Beach, FL). Before all experiments, flow cells were treated for 1 min with HBSA (HEK buffer with 1% bovine serum albumin (BSA)) and 1 min with 0.1 mg/mL streptavidin dissolved in HBSA. Flow cells were then equilibrated with 1× TIRF buffer (10 mM K^+^-imidazole, 50 mM KCl, 1 mM MgCl_2_, 1 mM EGTA, 0.2 mM ATP, 10 mM DTT, 15 mM glucose, 20 µg/mL catalase, 100 µg/mL glucose oxidase, and 0.5% methylcellulose (4000 cP), pH 7.4) before initiating each reaction. Solutions in TIRF chambers were exchanged using a syringe-pump (Harvard Apparatus, Holliston, MA) set at 60 µL/min flow rate. Time-lapse TIRF microscopy was performed using a Nikon-Ti200 inverted microscope (Nikon Instruments, Melville, NY) equipped with a MLC400 Monolithic Laser Combiner (Agilent Technology Inc., Santa Clara, CA), a TIRF-objective with a numerical aperture of 1.49 (Nikon Instruments), and an EMCCD camera (Andor Ixon, Belfast, Northern Ireland). The pixel size corresponded to either 0.27 µm × 0.27 µm or 0.18 µm × 0.18 µm (with 1.5× on-scope magnification). Unless noted otherwise, all reactions were acquired using 50 ms exposure. During recordings, focus was maintained using the Perfect Focus System (Nikon Instruments). All recorded movies were analyzed using ImageJ software within the FIJI suite^60^. Detection of single molecules was performed using spot detection software included in the Mosaic suite^61^. When manually analyzing single-molecule colocalization events, lifetimes of colocalization were determined by the number of frames in which fluorescence from both molecules overlapped spatially, multiplied by the acquisition intervals (e.g., in experiments where acquisition intervals were 1 s, the lifetime of a colocalization event lasting 3 frames was defined as 3 s).

To determine Abp1 effects on Arp2/3-mediated nucleation of daughter branches, actin (1 µM actin, 10% OG-labeled) was polymerized in the presence of 8 nM Arp2/3 complex, with or without 300 nM Abp1. Images were captured for 15 min at 10 s intervals. The emergence of daughter branches in each field of view (~20,000 µm^2^) was tracked and plotted as cumulative branching curves over time. The elongation rates of mother and daughter filaments in the reactions were measured using ImageJ by linearly extrapolating filament length over time.

Arp2/3 lifetime on actin filaments in the presence and absence of Abp1 was determined by visualizing JF646-SNAP-Arp2/3 molecule’s interaction with tethered actin filaments. Tethered filaments were first polymerized from 1 µM G-actin (10% OG-labeled, 1% biotin-labeled), washed with TIRF buffer, exposed to 10 nM JF646-SNAP-Arp2/3 with and without different concentrations of Abp1, and recorded at 0.15 s intervals. Lifetimes of SNAP-Arp2/3 on actin filaments were quantified in ImageJ and fitted with a single exponential probability distribution (without Abp1) or double exponential probability distribution (with Abp1) using maximum likelihood algorithm as previously described^62^. Standard errors for each parameter were determined using the bootstrap function in MATLAB (Mathworks, Natick, MA) with 10000 iterations.

For the single-molecule step photobleaching analysis shown in Fig. 2, 10 pM Abp1-SNAP-biotin-649 in TIRF buffer without glucose oxidase and catalase was transferred into a flow cell as above, and the immobilized spots (anchored by streptavidin-biotin-PEG linkage to the slide surface) were subjected continuous exposure. Fluorescence intensities of individual spots were obtained by measuring the mean signal of a 6 × 6 pixel box (~1.5 µm^2^) encompassing each spot in ImageJ. Stepwise reductions in the integrated fluorescence intensity time records of individual spots were identified and counted. For the step photobleaching analysis in Supplementary Figure 2, a higher concentration of Abp1 (15 nM) was passively absorbed to the glass surface, and otherwise the analysis was the same.

For TIRF experiments monitoring Abp1-SNAP binding to actin filaments, actin (1 µM final, 10% OG-actin and 0.5% biotin-actin) was polymerized and filaments were tethered to the glass slide as above. Next, the TIRF chamber was washed with excess 1× TIRF buffer, and then different concentrations of Abp1-SNAP-649 were flowed in. To visualize initial binding of Abp1-SNAP-649 to filaments, movies were recorded at 5 s intervals for 10 min, with *t* = 0 corresponding to the initiation of flow-in. Abp1-SNAP-649 intensities on filaments were measured as follows. Intensity of the 649 nm signal was integrated over a 3-pixel wide region along a filament, and background intensity was determined by integrating signal over a similar 3-pixel wide region next to the filament. The final Abp1-SNAP-649 intensity was calculated by subtracting background intensity from total intensity. Measurements were made at *t* = 300 s for 10 filaments at each concentration of Abp1-SNAP-649. Intensity profiles for Abp1-SNAP-649 accumulation on actin filaments over time were determined by integrating a 3-pixel wide region along actin filaments at different times.

For experiments measuring Arp2/3 binding to immobilized Abp1 molecules, Abp1-SNAP-549-Biotin was immobilized on the glass surface using the streptavidin-biotin linkage as above. Then, TIRF chambers were washed, 15 nM JF646-SNAP-Arp2/3 was flowed in, and images were acquired every 0.25 s for 1 min. Lifetimes of SNAP-Arp2/3 binding to Abp1-SNAP were measured from fluorescence trace of JF646-SNAP-Arp2/3 at target locations using ImageJ. Conversely, single-molecule experiments measuring free Abp1 binding to immobilized Arp2/3 complex were performed using 649-biotin-SNAP-Arp2/3 complex tethered by streptavidin-biotin linkage, and then flowing in 20 nM Abp1-SNAP-549 with or without 0.2 µM unlabeled Abp1. Images were acquired every 0.25 s for 2 min. Lifetimes of Abp1-SNAP-549 interactions with SNAP-Arp2/3 complex were measured as above.

To determine lifetimes of Abp1-SNAP molecules on branched filaments, 10 nM Abp1-SNAP-649, 8 nM Arp2/3 complex, and 1 µM G-actin (10% OG-labeled, 0.5% biotin-labeled) were mixed to polymerize actin, and images were captured at 1 s intervals for 10 min. Lifetimes of Abp1-SNAP molecules on filament sides or branch junctions were measured in ImageJ. Resulting lifetimes were fitted with a single exponential probability distribution (filament side binding) or double exponential probability distribution (branch junction binding) using maximum likelihood algorithm as previously described^62^. Standard errors for each parameter were determined using the bootstrap function in MATLAB (Mathworks) with 10,000 iterations. The frequencies of Abp1-SNAP binding to branch junctions, filament sides, or background (coverslip regions without visible F-actin) were determined by measuring the number of appearances of Abp1-SNAP molecules within a 1.14 µm^2^ box at each location over time and dividing this by the total amount of time when the detection box was available, i.e., unoccupied by Abp1-SNAP. Abp1-SNAP occupancies on filament sides and at branch sites were calculated as previously described^62^, assuming 370 binding sites/µm for F-actin side binding and 1 binding site/branch junction for branch binding.

Actin debranching assays were performed as follows. Branched actin filaments were polymerized and anchored using 1 µM actin (10% OG-labeled and 0.5% biotin-labeled), 10 nM GST-VCA, and 2 nM Arp2/3 complex, until the desired density of filaments was reached. Then, the assembly reaction was stopped by washing out soluble components. Next, different concentrations of Gmf1 and/or Abp1 were flowed in, and debranching was monitored for 15 min, capturing images at 10 s intervals. Branch lifetimes were measured in ImageJ by following the number of frames each branch persisted, and those data were used to generate cumulative branch survival curves.

### Pyrene-actin assembly assay

Gel-filtered monomeric actin in G-buffer was cleared by ultracentrifugation for 1 h at 4 °C at 350,000 × *g* in a TLA-100 rotor (Beckman Coulter Inc.), and the upper ~50% of the supernatant was carefully recovered and used for nucleation assays. All reactions (60 µL) contained 2 µM G-actin (10% pyrene labeled), which was converted to Mg^2+^-ATP-actin 2 min before use. Then, 42 µL Mg^2+^-ATP-G-actin was mixed rapidly with 15 µL proteins or control buffer and 3 µL of 20× initiation mix (40 mM MgCl_2_, 10 mM ATP, and 1 M KCl) to initiate the reactions. Pyrene-actin fluorescence was monitored for 3000 s using an Infinite M200 plate reader (Tecan Group Ltd, Zurich, Switzerland) at excitation and emission wavelengths of 365 and 407 nm, respectively. The baselines and plateaus of the resulting curves were corrected to each other, and arbitrarily set to 0 and 1, respectively. For reactions that did not reach a plateau (actin alone and Arp2/3+actin), the maximum value was assumed to be the average of those that did reach plateau.

### Analytical gel-filtration chromatography

Purifications of Arp2/3 complex, Gmf1, Gmf1-eGFP and Abp1 used in gel-filtration experiments are described above. All experiments were performed at 4 °C. Abp1 binding stoichiometry to Arp2/3 complex (Fig. 3g, h; and Supplementary Figure 3) was determined using a Superdex 200 Increase 10/300 GL column run on an Akta Pure M liquid chromatography system (GE Healthcare). The Akta system was equipped with a 10 mm flow cell and monochromator-based wavelength selection for absorbance detection, allowing total protein in the presence of 0.1 mM ATP to be followed at 290 nm. Samples were prepared by diluting Arp2/3 complex (to 700 nM) and/or Abp1 (to 7 µM) from freshly gel-filtered stocks, bringing the final volume to 0.56 mL with gel-filtration buffer (10 mM HEPES pH 7.0, 50 mM potassium chloride, 1 mM EGTA pH 8.0, 2 mM MgCl_2_, 0.5 mM DTT, and 0.1 mM ATP), incubating for >1 h on ice, and then loading into 0.5 mL capillary loops. Stock concentrations were measured by ultraviolet absorbance. Samples were applied to the column, which was run at a flow rate of 0.35 mL/min in gel-filtration buffer, and 0.65 mL fractions were collected. Select fractions were run on Criterion TGX Stain-Free gels (Bio-Rad Laboratories, Hercules, CA), imaged for ‘Stain-Free’ signal using a Gel Doc EZ Imager, and bands were picked and quantified using ImageLab software (Bio-Rad). Standards for Arp2/3 complex and Abp1 proteins were prepared from the same stocks used for complex formation, and were diluted to 1 µM (Arp2/3 complex) or 2 µM (Abp1), from which a serial twofold dilution series was prepared. To ensure consistent quantitation, standards and samples to quantify were run and imaged on the same 26-lane gel. Background intensity was calculated from an adjacent empty lane and subtracted in the analysis.

Gmf1-eGFP competition experiments (Fig. 5b, c) were performed using Superdex 200 PC (3.2/30) column run in a 30 cm precision column holder (GE-Healthcare) on an Ettan microscale protein liquid chromatography system (Amersham Pharmacia, Buckinghamshire, UK). The system was equipped with monochrometer-based wavelength selection for absorbance detection, allowing Gmf1-eGFP to be followed by its absorbance at 492 nm. Samples were applied to the column in a total loading volume of 100 µL using a Hamilton syringe. The column was run at a flow rate of 50 µL/min in 10 mM HEPES pH 7.5, 50 mM potassium chloride, 1 mM EGTA pH 8.0, 2 mM MgCl_2_, 0.5 mM DTT, and 0.1 mM ATP.

### Single-particle electron microscopy

To image Abp1-SNAP bound to Arp2/3 complex, the two proteins were incubated together in a 1:1 molar ratio (100 nM each) for 30 min at room temperature, then applied to carbon-coated glow-discharged grids, negatively stained with 0.75% Uranyl formate for 30 s, air-dried, and imaged using a JEOL 2100 transmission electron microscope at 200 kV (low-dose conditions). Images were captured using an Ultrascan 1000XP CCD camera (Gatan Inc., Pleasanton, CA) at 40,000× magnification and 1.5–2.8 μm underfocus (see Supplementary Table 1 for relevant information on data collection and refinement). Briefly, a total of 968 pairs of particles, untilted and tilted at 45 degrees, were collected using BOXER. Preliminary 3D reconstructions were obtained using random conical tilt method, implemented in EMAN 2.1. For refinement of the preliminary model, we used EMAN 2.1 and RELION 2.0^63,64^. An additional 4328 untilted views of Abp1–Arp2/3 complexes were collected using BOXER. Correction for the contrast transfer function of the microscope was done in EMAN 2.1. The 6264 particles (968 tilted and 5296 untilted) were classified using RELION 2.0 into 25 classes. Of these particles, 508 were discarded due to poor quality, based on assessment using an algorithm implemented in RELION 2.0. The remaining 5756 particles were placed in new sets according to grouping into different 3D classes, yielding one reconstruction of unbound Arp2/3 complex and two reconstructions of Abp1-bound Arp2/3 complex. The resolution of the reconstructions was determined by Fourier shell correlation: unbound Arp2/3 complex (18 Å), Class 1 and Class 2 Abp1-bound Arp2/3 complexes (21 Å). 3D difference mapping was performed using UCSF Chimera package (developed by the Resource for Biocomputing, Visualization, and Informatics at the University of California, San Francisco (supported by NIGMS P41-GM103311)). For this, we used the crystal structure of Arp2/3 complex (PDB: 4XF2) and calculated its 3D structure at 20 Å resolution using command *molmap*, then using command *vop subtract* we produced the difference maps between low-resolution Arp2/3 crystal structure and Arp2/3-Abp1 complexes.

### Statistical information

All error bars are defined throughout the figures. All figures were produced in Graphpad Prism 6.0.

### Data availability

Data supporting the findings of this study are available from the corresponding author upon reasonable request. 3D reconstructions of Abp1–Arp2/3 complex have been deposited to EMDB: open Arp2/3 complex (EMD-4267), Class 1 Abp1–Arp2/3 complex (EMD-4269), and Class 2 Abp1–Arp2/3 complex (EMD-4268).

## Electronic supplementary material

Supplementary Information
