# Supplementary material for: Abp1 promotes Arp2/3 complex-dependent actin nucleation and stabilizes branch junctions by antagonizing GMF
Source: Nat Commun. 2018 Jul 24;9:2895. doi: 10.1038/s41467-018-05260-y (PMC6057921; doi:10.1038/s41467-018-05260-y)
Supplement: Supplementary file 1 — Supplementary Information [file 41467_2018_5260_MOESM1_ESM.pdf]

Abp1 promotes Arp2/3 complex-dependent actin nucleation and stabilizes branch junctions by antagonizing GMF

Guo *et al.*

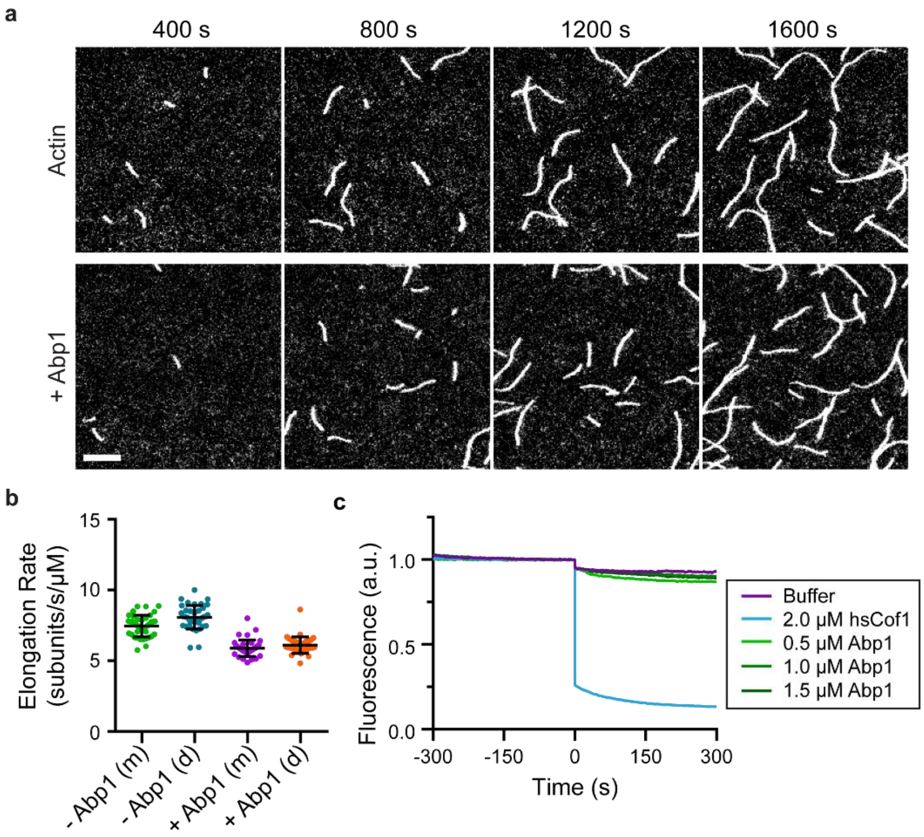

Supplementary Figure 1. Abp1 alone controls for effects on actin filament dynamics and quenching of pyrene-F-actin fluorescence. (a) Time points from representative TIRF microscopy actin assembly assays. Reactions contain 1 µM actin (10% OG-labeled) with and without 300 nM Abp1. Scale bar, 10 µm. **(b)** Actin filament elongation rates for mother (m) and daughter (d) filaments in TIRF reactions containing Arp2/3 complex, as in figure 1a and 1b, measured for n = 40 filaments per condition. Mean with s.d. **(c)** Actin (2 µM, 10% pyrene-labeled) was polymerized to steady state, and then fluorescence was monitored over time. The 'zero' time point indicates where buffer and/or the indicated concentrations of proteins (hsCof1 or Abp1) were added. HsCof1 strongly quenched pyrene fluorescence, as previously reported[1], whereas Abp1 did not.

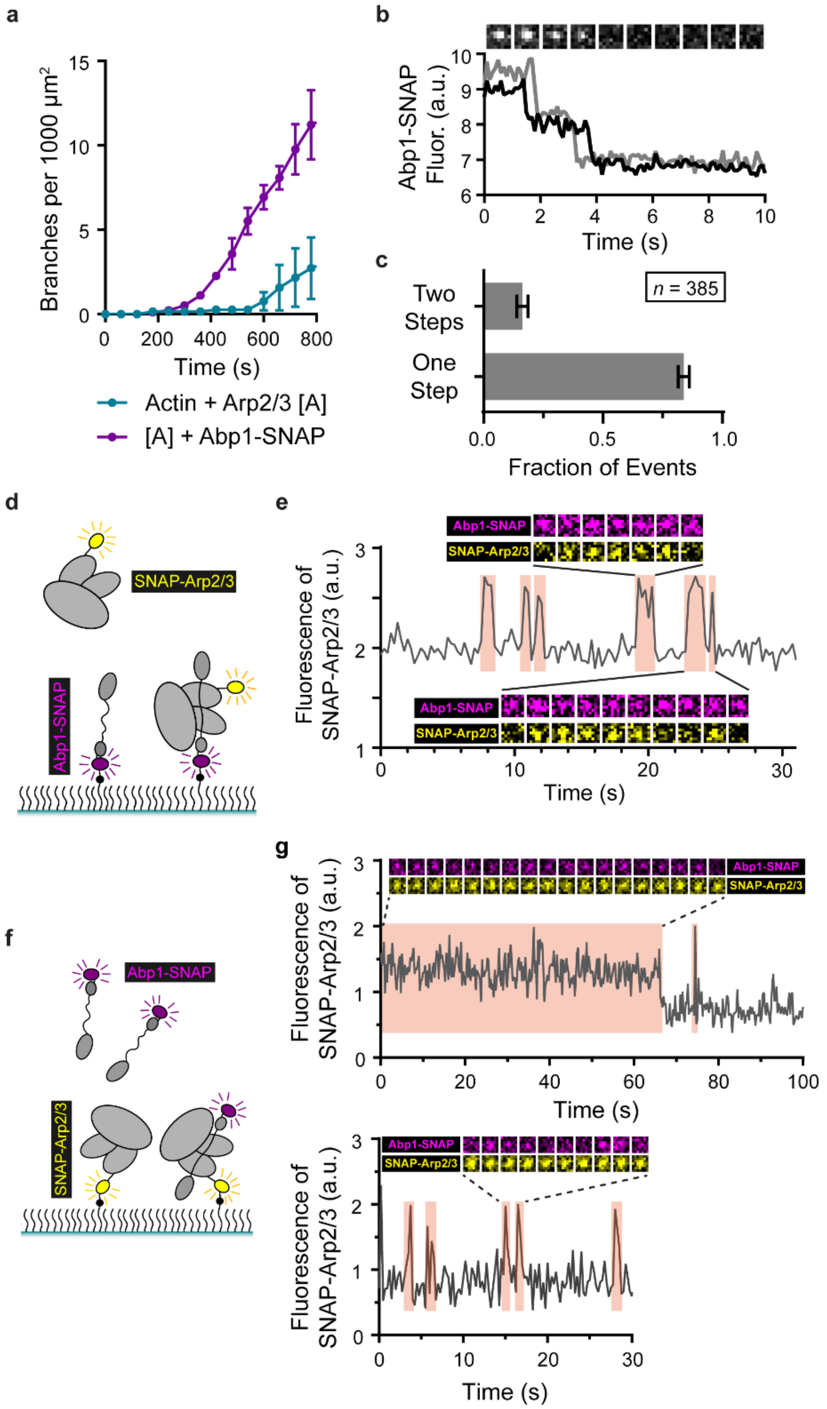

Supplementary Figure 2. Analysis of Abp1-SNAP. (a) Arp2/3-mediated branch nucleation in TIRF assays containing 1 µM actin (10% OG-labeled) and 8 nM Arp2/3 complex, with or without 300 nM Abp1-SNAP-649. Kinetics of branch formation, quantified as the number of branches nucleated per 1000 µm^2, averaged from 2 trials. Error bars, s.e.m. **(b)** Representative step photobleaching of passively-absorbed Abp1-SNAP-649 molecules. Plot shows fluorescence intensity over time for two spots that bleached in two steps. Inset shows montage images of one representative two-step photobleaching event. **(c)** Fraction of Abp1-SNAP-649 molecules (15 nM) that photobleached in one versus two steps from analysis as in (A) for a total of *n* = 385 spots from 2 separate trials. Error bars, s.e.m. **(d)** Schematic of experimental setup for measuring interactions between surface tethered labeled Abp1 molecules and labeled free Arp2/3 complex molecules (as seen in Figure 3a-c). **(e)** Example time record of JF646-SNAP-Arp2/3 molecules (*yellow*) binding and dissociating from one surface-tethered Abp1-SNAP-biotin-649 molecules (*magenta*). Shaded intervals (*rose*) indicate the presence of JF646-SNAP-Arp2/3 fluorescence. Inset montage (2.1 × 2.1 µm) shows 0.25 s intervals. **(f)** Schematic of experimental setup for measuring interactions between surface tethered labeled Arp2/3 complex molecules and labeled Abp1 molecules (as seen in Figure 3d-f). **(g)** Two example time recordings of binding events (highlighting both long-lived and short-lived interactions) between Abp1-SNAP-549 molecules (*magenta*) and surface-tethered 649-biotin-SNAP-Arp2/3 molecules (*yellow*) in the presence of 0.2 µM unlabeled Abp1. Shaded time intervals (*rose*) indicate the presence of Abp1-SNAP-549 fluorescence. Top inset montage (1.5 µm × 1.5 µm) shows 4 s intervals. Bottom inset montage (1.5 µm × 1.5 µm) shows 0.25 s intervals.

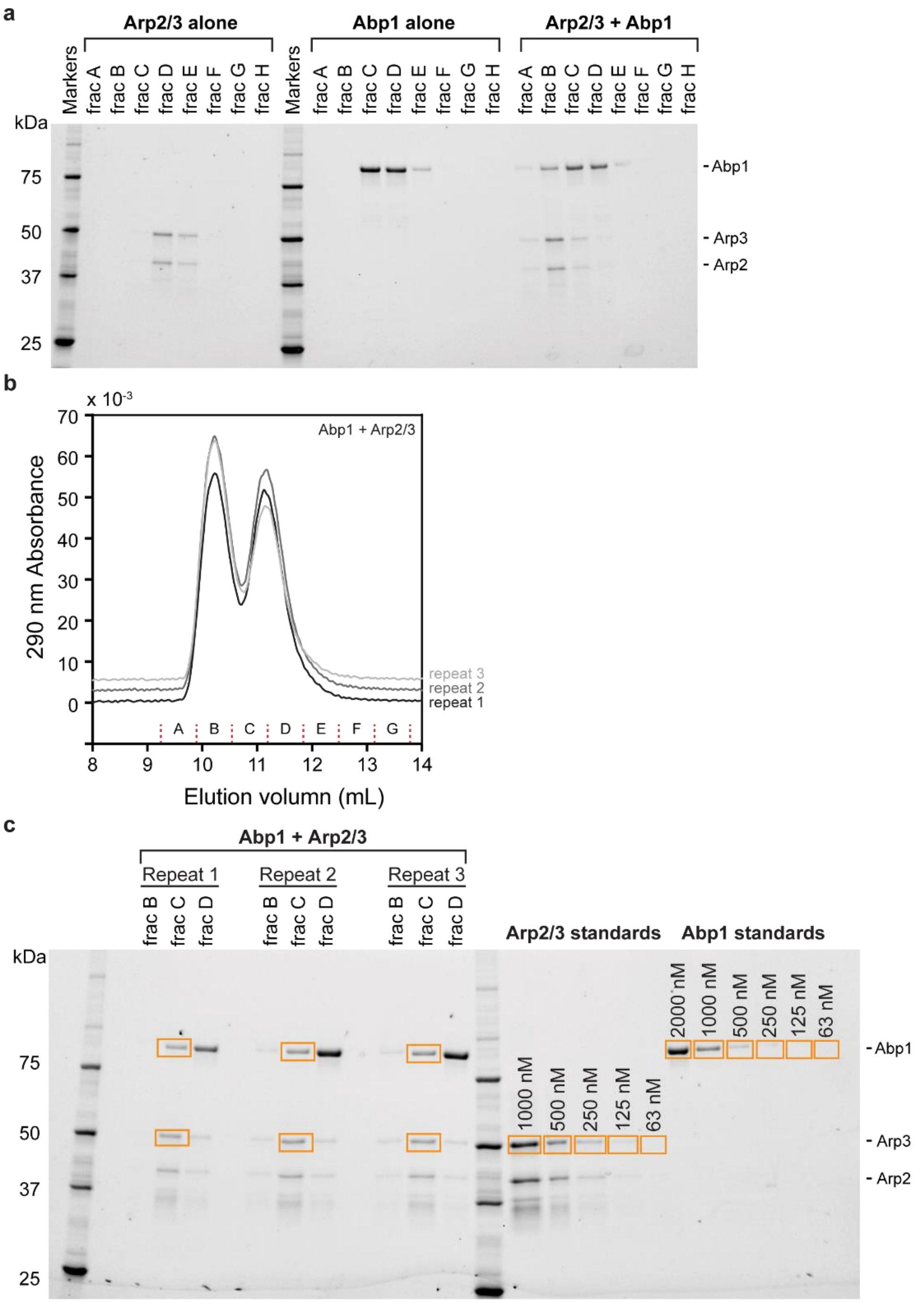

Supplementary Figure 3. Size exclusion chromatography analysis of Abp1-Arp2/3 complex binding stoichiometry. (a) Full SDS-PAGE 'stain-free' gels showing fractions from gel filtration analysis of Arp2/3 complex alone, Abp1 alone, and the mixture of Abp1 and Arp2/3 complex (crops of same gel shown in Figure 3g). **(b)** Size exclusion chromatograms of Abp1-bound Arp2/3 complex observed in three repeat trials (repeat 1 is shown in Figure 3g). **(c)** Quantitative analysis of bands on stain-free gels from the three repeat trials, with serial dilutions of purified proteins as standard curves, used to estimate the stoichiometry of Abp1 binding to Arp2/3 complex (see table in Figure 3h).

SUPPLEMENTARY METHOD

Pyrene F-actin quenching assay

Actin (10% pyrene-labeled) was polymerized to steady state in F-buffer (10 mM Tris-HCl pH 7.5, 0.2 mM DTT, 0.2 mM CaCl2, 50 mM KCl, 2 mM MgCl2, and 0.7 mM ATP) for 2 hr at 25ºC. Pyrene signal was monitored at 25ºC in a fluorescence spectrophotometer (Photon Technology International, Lawrenceville, NJ) at excitation 365 nm and emission 407 nm. Fluorescence of 2 µM pyrene-F-actin was monitored for 300 s before spiking in buffer (HEK buffer), hsCof1, or Abp1. Changes in fluorescence were then monitored for 300 s. Resulting fluorescence curves were shifted so that 0 s corresponds to time of spike-in, and the average fluorescence between -30 s to 0 s was taken to be value of one. Human Cof1 was purified precisely as described[2].

SUPPLEMENTARY REFERENCES

1. Blanchoin, L. & Pollard, T. D. Interaction of Actin Monomers with AcanthamoebaActophorin (ADF/Cofilin) and Profilin. *J. Biol. Chem.* **273,** 25106–25111 (1998).
2. Jansen, S. *et al.* Single-molecule imaging of a three-component ordered actin disassembly mechanism. *Nat. Commun.* **6,** 7202 (2015).
